# Targeting BCL‐2 with venetoclax and dexamethasone in patients with relapsed/refractory t(11;14) multiple myeloma

**DOI:** 10.1002/ajh.26083

**Published:** 2021-01-19

**Authors:** Jonathan L. Kaufman, Cristina Gasparetto, Fredrik H. Schjesvold, Philippe Moreau, Cyrille Touzeau, Thierry Facon, Lawrence H. Boise, Yanwen Jiang, Xiaoqing Yang, Fengjiao Dunbar, Deeksha Vishwamitra, Stefanie Unger, Tammy Macartney, John Pesko, Yao Yu, Ahmed Hamed Salem, Jeremy A. Ross, Wan‐Jen Hong, Paulo C. Maciag, James M. Pauff, Shaji Kumar

**Affiliations:** ^1^ Winship Cancer Institute of Emory University Atlanta Georgia USA; ^2^ Duke University, Hematologic Malignancies & Cellular Therapy Durham North Carolina USA; ^3^ Oslo Myeloma Center, Oslo University Hospital, Oslo, Norway and K.G. Jebsen Center for B‐cell malignancies, University of Oslo Oslo Norway; ^4^ University Hospital, Nantes, France CRCINA, INSERM, Centre National de la Recherche Scientifique, University of Angers, University of Nantes Nantes France; ^5^ Centre Hospitalier Regional Universitaire Lille, Hospital Huriez Lille France; ^6^ Genentech Inc. South San Francisco California USA; ^7^ AbbVie Inc North Chicago Illinois USA; ^8^ AbbVie Deutschland GmbH & Co KG Wiesbaden Germany; ^9^ Mayo Clinic Rochester Minnesota USA

## Abstract

Venetoclax (Ven) is a selective small‐molecule inhibitor of BCL‐2 that exhibits antitumoral activity against MM cells with t(11;14) translocation. We evaluated the safety and efficacy of Ven and dexamethasone (VenDex) combination in patients with t(11;14) positive relapsed/refractory (R/R) multiple myeloma (MM). This open‐label, multicenter study had two distinct phases (phase one [P1], phase two [P2]). Patients in both phases received VenDex (oral Ven 800 mg/day + oral Dex 40 mg [20 mg for patients ≥75 years] on days 1, 8, and 15, per 21–day cycle). The primary objective of the P1 VenDex cohort was to assess safety and pharmacokinetics. Phase two further evaluated efficacy with objective response rate (ORR) and very good partial response or better. Correlative studies explored baseline *BCL2* (BCL‐2) and *BCL2L1* (BCL‐X_L_) gene expression, cytogenetics, and recurrent somatic mutations in MM. Twenty and 31 patients in P1 and P2 with t(11;14) positive translocation received VenDex. P1/P2 patients had received a median of 3/5 lines of prior therapy, and 20%/87% were refractory to daratumumab. Predominant grade 3/4 hematological adverse events (AEs) with ≥10% occurrence included lymphopenia (20%/19%), neutropenia (15%/7%), thrombocytopenia (10%/10%), and anemia (5%/16%). At a median follow‐up of 12.3/9.2 months, ORR was 60%/48%. The duration of response estimate at 12 months was 50%/61%, and the median time to progression was 12.4/10.8 months. In biomarker evaluable patients, response to VenDex was independent of concurrent del(17p) or gain(1q) and mutations in key oncogenic signaling pathways, including MAPK and NF‐kB. VenDex demonstrated efficacy and manageable safety in heavily‐pre‐treated patients with t(11;14) R/R MM.

## 1 INTRODUCTION

Multiple myeloma (MM) is an incurable disease that is heterogeneous in clinical presentation, responsiveness to therapy, and long‐term survival, including variations in the underlying chromosomal abnormalities.^1^ Recent advances in treatment including the development of proteasome inhibitors (PI), immunomodulatory drugs (IMiD), and monoclonal antibodies have contributed to improved overall and event‐free survival periods; however, patients eventually relapse and become increasingly refractory to currently available therapies resulting in successively shorter remissions.^2^, ^3^, ^4^, ^5^, ^6^

The BCL‐2 family of proteins is essential in the regulation of apoptosis and cell survival. BCL‐2, MCL‐1, and BCL‐X_L_ are anti‐apoptotic proteins of the BCL‐2 family that promote MM cell survival. MM is heterogeneous with respect to BCL‐2 family dependency, with some cases being more dependent on MCL‐1 over BCL‐2 and vice versa.^7^ Thus, t (11;14) is the most common chromosome translocation in MM with an occurrence rate of 15% – 20%.^8^, ^9^ Studies in human myeloma cell lines have demonstrated that the presence of t(11;14) is predictive of BCL‐2 dependency.^10^, ^11^ Venetoclax (Ven) is a potent, selective, orally bioavailable inhibitor of BCL‐2. Selective targeting of BCL‐2 with Ven has shown promising antitumor activity in several hematologic malignancies, including chronic lymphocytic leukemia, acute myeloid leukemia, and non‐Hodgkin lymphomas. in vitro data showed a high sensitivity to Ven in human myeloma cell lines and primary MM samples that were positive for the t(11;14) translocation.^12^ Additionally, the sensitivity to BCL‐2 inhibition in the t(11;14) subset was associated with higher expression of BCL‐2 than MCL‐1 or BCL‐X_L._ ^7^, ^11^

We have previously shown that Ven demonstrated promising single‐agent activity in patients with t(11;14) positive relapsed/refractory (R/R) MM, with 40% objective response rate (ORR) and 27% achieving at least a very good partial response or better (≥VGPR).^13^ Response to Ven monotherapy also correlated with a higher *BCL2:BCL2L1* gene expression ratio, indicating *BCL2L1* (BCL‐X_L_) may be a key resistance factor to broader Ven activity within the t(11;14) subgroup.^13^ Preclinical studies in MM cell lines and primary patient samples have demonstrated that dexamethasone (Dex) used in combination with Ven can significantly increase cell death compared to Ven alone.^14^ Treatment of MM cells with Dex increases expression of BCL‐2 as well as pro‐apoptotic proteins BIM and PUMA while decreasing the expression of BCL‐X_L_.^14^, ^15^, ^16^, ^17^, ^18^, ^19^ Thus, Dex is hypothesized to induce “BCL‐2 priming”, a state where BCL‐2 maintains cell survival by sequestering high levels of BIM, providing a rationale for use as a combination agent with Ven in MM.^14^

Here, we report the efficacy and safety of the VenDex combination from a phase 1/2 study as a therapeutic approach to improve clinical outcomes in patients with t(11;14) R/R MM. Outcomes by baseline *BCL2* and *BCL2L1* gene expression, cytogenetic abnormalities concurrent with t(11;14), and somatic mutations recurrent in MM were also explored.

## 2 METHODS

### 2.1 Study design

This open‐label phase 1/2 study (NCT01794520) had two distinct phases; phase one and phase two (Figure S1). Phase one included dose‐escalation, safety expansion, and a VenDex combination. The primary objectives of phase one were to assess the safety profile, characterize pharmacokinetics (PK), determine the dosing schedule, the maximum tolerated dose, and the recommended phase two dose of venetoclax monotherapy when administered in patients with R/R MM. In addition, the safety and PK profiles of t(11;14) positive patients treated with VenDex was also evaluated. The secondary objectives were to evaluate the preliminary efficacy of Ven monotherapy or VenDex on ORR, time to response (TTR), time to disease progression (TTP), and duration of response (DoR). Patients discontinued the study treatment if they had disease progression, toxicity or intolerability, and were followed for safety through the treatment‐emergent period (ie, 30 days after the discontinuation of study drug). Here, we present data from patients with t (11;14) positive R/R MM from the safety‐expansion cohort who received VenDex.

Phase two was an expansion cohort of the VenDex combination that further evaluated the efficacy of the combination in R/R patients with t(11;14) positive MM. The primary objectives evaluated were ORR and a very good partial response or better (≥VGPR). Secondary objectives included safety, progression‐free survival (PFS), TTR, TTP, DoR, and overall survival (OS). Patients discontinued treatment upon disease progression and were then followed for OS. Patients who discontinued study treatment for reasons other than disease progression were monitored for disease progression and followed for OS.

The data cut‐off for this publication was September 2, 2019. The trial was conducted under the International Conference on Harmonization Good Clinical Practice guidelines and according to the Declaration of Helsinki. A local institutional review board or ethics committee approved the study at each site. All patients provided written informed consent before participation.

### 2.2 Patient enrollment and treatment

Detailed eligibility criteria were previously published.^13^ In brief, eligible patients were adults with R/R MM ≥18 years of age. In addition, patients included in the phase one VenDex combination cohort were t(11;14) positive as determined by the fluorescence in‐situ hybridization (FISH) assay per central laboratory and had received at least one prior treatment with a PI and an IMiD, and had an Eastern Co‐operative Oncology Group (ECOG) score ≤ 1.

Patients enrolled in the phase two VenDex cohort had t(11;14) positive MM as determined by the FISH assay per central laboratory testing or were enrolled at the discretion of the investigator if the FISH assays were performed at a local laboratory. The patients have had received at least two prior lines of therapy, including a PI, IMiD, daratumumab, and glucocorticoids, with evidence of disease progression on or within 60 days of the last dose of the most recent line of treatment based on the International Myeloma Working Group (IMWG) criteria. For all patients enrolled in the United States, daratumumab combination therapy was required as one of the prior lines of therapy. For patients enrolled outside the United States, either daratumumab monotherapy (restricted to 20% of total enrolled patients) or combination therapy was acceptable.

In both phases, patients received VenDex combination with daily oral doses of Ven 800 mg and Dex 40 mg on days 1, 8, and 15 of each 21– day cycle. Patients above 75 years of age started Dex at a 20 mg dose.^13^ The optimal dose of 800 mg of Ven was selected based on an exploratory exposure‐response analyses based on PK, best IMWG responses in t(11;14) positive patients, and safety (≥ grade three anemia, thrombocytopenia, and neutropenia). This analysis also found that increasing the Ven dose to 1200 mg may increase the response rates marginally; however, the reduced dose intensities at 1200 mg resulted in more inter‐patient Ven exposure variability and indicated reduced compliance at the highest doses. This suggested that a higher dose of Ven was sub‐optimal for long‐term therapy.

### 2.3 Study assessments

#### 2.3.1 Safety

Assessments were conducted throughout the study. Adverse events (AEs) were graded according to the National Cancer Institute Common Terminology Criteria for Adverse Events (version 4.0).^20^

#### 2.3.2 Efficacy

In phase one, the efficacy of VenDex was assessed as the ORR, TTP, and DoR based on the 2011 International Uniform Response Criteria for MM.^21^ Efficacy in phase two further evaluated ORR and ≥ VGPR using the 2016 IMWG response criteria.^22^ The OS was also assessed.

#### 2.3.3 Exploratory biomarkers

Bone marrow aspirate specimens were collected at baseline for interphase FISH analysis on cluster of differentiation (CD)138‐enriched bone marrow mononuclear cells (BMMCs) using probes for t(11;14), del(17p), gain(1q), and chromosomes (Ch) 5, 9, or 15. The threshold for determining positivity for FISH was based on the analytical cut off determined for each probe. Hyperdiploidy was defined as the presence of polysomy Ch5, Ch9, or Ch15, as detected by FISH analysis. Expression of *BCL2* (BCL‐2) and *BCL2L1* (BCL‐X_L_) mRNA in CD138‐enriched BMMCs was determined by qPCR using the ΔCt (cycle threshold) method. Baseline CD138‐enriched BMMC samples were also evaluated for somatic mutations by whole‐exome sequencing. Sequencing analysis was performed using a variant detection threshold of 10% and read depths of >10 for single nucleotide variant calls. Putative germline variants found in population databases (dbSNP, ExAC) were removed from the analysis unless known to be cancer‐related (COSMIC) or predicted to be deleterious (FATHMM‐MKL). Highly recurrent genetic alterations in MM across a range of functional classifications, including cell signaling, cell cycle, DNA repair, and plasma cell differentiation, were analyzed within the study cohorts. Due to the small sample size of biomarker populations, the results of the two cohorts were combined.

### 2.4 Statistical analysis

All t(11;14) positive patients who received at least one dose of Ven were included in the safety and efficacy analyses. Descriptive statistics, including medians, standard deviations, and ranges, were calculated. Kaplan–Meier methodology was used for time‐to‐event analyses.

## 3 RESULTS

### 3.1 Patient demographics and clinical characteristics

At data cut‐off, 20 and 31 patients with t(11;14) positive disease were enrolled in phase one and phase two VenDex cohorts, respectively. The median age of patients in phase one VenDex was 63 years (range: 46–77), and in phase two VenDex was 65 years (range: 48–80). The median number of prior lines of therapy received was three (range: 1–8) and five (range: 2–12) in phase one VenDex and phase two VenDex, respectively. In phase one/phase two VenDex, 25%/100% were exposed to prior daratumumab therapy of which 20%/87% were refractory to the daratumumab therapy. Key demographic and clinical characteristics are summarized in Table 1.

**TABLE 1:** Patient demographics and clinical characteristics

Characteristics	Phase 1 VenDex (N = 20)	Phase 2 VenDex (N = 31)
Age, median (range)	63 (46–77)	65 (48–80)
Male, *n* (%)	17 (85)	18 (58)
ISS stage, *n* (%)		
Stage 1	9 (47)	8 (28)
Stage 2	7 (37)	6 (21)
Stage 3	3 (16)	5 (17)
Not evaluable	0	10 (35)
Missing^a^	1	2
Chromosomal Abnormalities (CAs), *n* (%)		
del(17p)	2 (10)	8 (26)
gain(1q)	2 (10)	15 (48)
Hyperdiploid	5 (25)	9 (29)
No. of prior lines of therapy, median (range)	3 (1–8)	5 (2–12)
Stem cell transplant, *n* (%)	17 (85)	18 (58)
Exposed to prior PI, *n* (%)	20 (100)	31 (100)
Refractory to prior PI, *n* (%)	13 (65)	27 (87)
Exposed to prior IMiD, *n* (%)	20 (100)	30 (97)
Refractory to prior IMiD, *n* (%)	18 (90)	27 (87)
Exposed to prior PI+IMiD, *n* (%)	20 (100)	30 (97)
Refractory to prior PI+IMiD, *n* (%)	13 (65)	18 (58)
Exposed to prior daratumumab, *n* (%)	5 (25)	31 (100)
Refractory to prior daratumumab, *n* (%)	4 (20)	27 (87
indent left as this is a new row Refractory to last prior therapy, *n* (%)	17 (85)	30 (97)^b^

Abbreviations: IMiD, Immunomodulatory drug; ISS, International Staging System; PI, proteasome inhibitor; VenDex, Venetoclax plus Dexamethasone combination therapy.

^a^ Data not reported from clinical sites at the time of data cut‐off.

^b^ In one patient, last prior therapy outcome was not reported at the time of data cut‐off; however, study enrollment and dosing occurred ≤ 60 days from last therapy stop date.

### 3.2 Patient disposition

In phase one VenDex, 19 (95%) patients discontinued treatment of which 18 had progressive disease, and one underwent a transplant. The median time on study (ToS) was 12.9 (range: 1.1–34.6) months with a Kaplan–Meier estimated median follow‐up time of 12.3 months (95% CI: 5.4–22.6). No deaths were reported in the treatment‐emergent period.

In phase two VenDex, 10 (32%) patients were on treatment and 21 (68%) had discontinued treatment (18 progressive disease; one adverse event; one physician's decision; one other). Twelve (39%) patients were in follow‐up at the time of data cut‐off. Median ToS was 5.8 (range: 1.0–17.2) months, and the estimated median follow‐up time was 9.2 months (95% CI: 6.3–11.1). Eleven (36%) deaths were reported, of which eight were due to progressive disease, one unknown and two due to treatment‐emergent adverse events (one due to sepsis in the first month of study without confirmed progression, one due to bacterial abscess).

### 3.3 Safety

Most patients (96%) treated with VenDex experienced at least one treatment‐emergent adverse event (TEAE) (Table 2). In phase one VenDex, predominant hematological TEAEs with ≥20% occurrence included thrombocytopenia (30%), leukopenia (25%), lymphopenia (20%), and neutropenia (20%). The most frequent non‐hematological TEAEs with ≥30% occurrence reported were insomnia (45%), hypophosphatemia (40%), diarrhea (35%), hyperglycemia (35%), nausea (30%), and upper respiratory tract infection (30%). The most common grade three or four hematological TEAEs with ≥15% occurrence were lymphopenia (20%) and neutropenia (15%). The most common grade three or four non‐hematological AE with ≥15% occurrence was hypophosphatemia (20%). Infections were predominantly low grade, and included upper respiratory tract infection (RTI, 30%), bronchitis, sinusitis, and *Clostridium difficile* infection (10% each). Only one case of *C. difficile* was considered to be of grade three or four severity (Figure S2).

**TABLE 2:** Summary of treatment‐emergent adverse events [Color figure can be viewed at wileyonlinelibrary.com]

	Phase 1VenDex (N = 20)n (%)	Phase 2 VenDex (N = 31), n(%)	All VenDex (*N* = 51)n (%)
**Any treatment‐emergent adverse event (TEAE)**	**20 (100)**	**29 (94)**	**49 (96)**
Hematological			
Lymphopenia	4 (20)	10 (32)	14 (28)
Anemia	3 (15)	7 (23)	10 (20)
Neutropenia	4 (20)	5 (16)	9 (18)
Thrombocytopenia	6 (30)	3 (10)	9 (18)
Leukopenia	5 (25)	3 (10)	8 (16)
Non‐hematological			
Diarrhea	7 (35)	11 (36)	18 (35)
Nausea	6 (30)	8 (26)	14 (28)
Insomnia	9 (45)	4 (13)	13 (26)
Hyperglycemia	7 (35)	5 (16)	12 (24)
Hypophosphatemia	8 (40)	3 (10)	11 (22)
Cough	5 (25)	5 (17)	10 (20)
Upper respiratory tract infection	6 (30)	3 (10)	9 (18)
Blood creatinine increased	4 (20)	4 (13)	8 (16)
Blood lactase dehydrogenase increased	4 (20)	4 (13)	8 (16)
Fatigue	2 (10)	5 (16)	7 (14)
Hypokalemia	4 (20)	3 (10)	7 (14)
Arthralgia	4 (20)	3 (10)	7 (14)
Nasal congestion	5 (25)	2 (7)	7 (14)
Oropharyngeal pain	5 (25)	2 (7)	7 (14)
Headache	4 (20)	1 (3)	5 (10)
Pyrexia	4 (20)	0	4 (8)
Any TEAE grade 3 or 4	**14 (70)**	**21 (68)**	**35 (69)**
Hematological			
Lymphopenia	4 (20)	6 (19)	10 (20)
Anemia	1 (5)	5 (16)	6 (12)
Neutropenia	3 (15)	2 (7)	5 (10)
Thrombocytopenia	2 (10)	3 (10)	5 (10)
Non‐hematological			
Hypophosphatemia	4 (20)	1 (3)	5 (10)
Sepsis	1 (5)	3 (10)	4 (8)
Tumor lysis syndrome	2 (10)	1 (3)	3 (6)
Hyperuricemia	2 (10)	0	2 (4)
Any Serious AE	**6 (30)**	**13 (42)**	**19 (37)**
Sepsis	1 (5)	3 (10)	4 (8)
Tumor lysis syndrome	2 (10)	1 (3)	3 (6)
*Clostridium difficile*	1 (5)	1 (3)	2 (4)
Hypertension	0	2 (6)	2 (4)
Nausea	1 (5)	0	1 (2)
Vomiting	1 (5)	0	1 (2)

*Note*: Any TEAE included ≥20% of occurrence in either VenDex cohort. Grade 3 or 4 TEAEs included AEs ≥10% of occurrence in any VenDex cohort. Serious AEs included AEs ≥5% of occurrence in any VenDex cohort.

In phase two VenDex, the most frequent hematological TEAEs with ≥20% occurrence were lymphopenia (32%) and anemia (23%). The most frequent non‐hematological TEAE with ≥30% occurrence was diarrhea (36%). Predominant grade three or four TEAEs with ≥15% occurrence were lymphopenia (19%) and anemia (16%). Sepsis was the most commonly occurring infection, and all three cases (10%) were considered serious with one fatal outcome (sepsis without confirmed progression). Other common infections (any grade) were parainfluenza and upper RTI (10% each).

### 3.4 Efficacy

**FIGURE 1:**
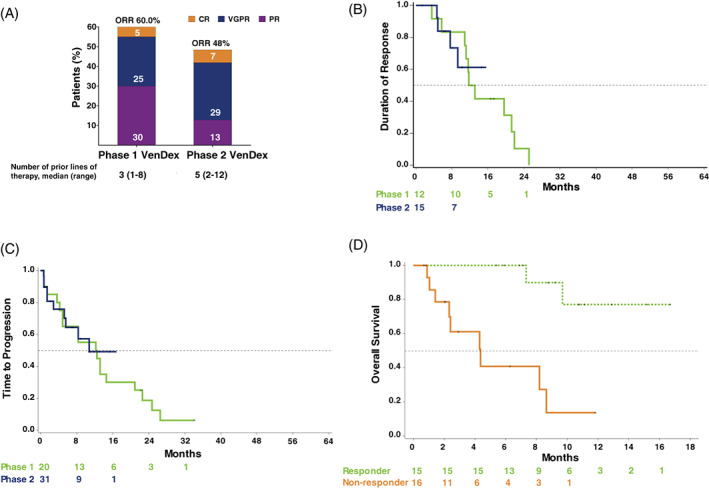
(A) Overall response rate by cohorts. ORR indicates a response of PR or better. ORR: Objective Response Rates; CR: Complete response; VGPR: Very good partial response; VenDex: Venetoclax and dexamethasone combination therapy; PR: Partial response. (B) Duration of response. (C) Time to progression. (D) Overall survival of patients in phase two venetoclax plus dexamethasone cohort stratified by patient response status (responders: patients with a partial response or better; non‐responder: patients without a minimum of a partial response). DoR: duration of response; OS: overall survival; TTP: time to progression

Responses varied by treatment group (Figure 1A). In phase 1 VenDex, median TTR was 1.4 months (range: 0.7–5.7), and ORR was achieved by 12 patients (60%), with ≥VGPR achieved by six (30%) patients. Complete response (CR) was observed in one (5%), VGPR in five (25%), and partial response (PR) was observed in six (30%) patients. The Kaplan–Meier estimates for median DoR and TTP were 12.4 months (95% CI: 5.7–21.2) and 12.4 months (95% CI: 4.2–20.9), respectively (Figure 1B,C).

Median TTR in phase two VenDex was 0.7 months (range: 0.6–1.5), and ORR was achieved by 15 patients (48%), with ≥VGPR achieved by 11 (36%) patients. Complete remission was observed in two (7%), VGPR in nine (29%), and PR was observed in four (13%) patients. The median DoR was not yet estimable via Kaplan–Meier methodology. The estimate of DoR at 12‐months was 61% (95% CI: 25% – 84%), and the estimated median TTP was 10.8 months (range: 5.2 – not reached). The 12‐month estimate of OS for responders (Figure 1D) was 77% (95% CI: 35% – 94%).

### 3.5 Exploratory biomarkers

Of the 51 t(11;14) R/R MM patients who received VenDex, 10 (20%) and 17 (33%) had concurrent del(17p) or gain(1q), respectively. Of note, the majority of patients with del(17p) or gain(1q) were in the phase two cohort of the study. In patients with del(17p), ORR was achieved by five (50%) and ≥ VGPR was achieved by two (20%) patients. In patients with gain(1q), ORR was achieved by 7/17 (41%) and ≥ VGPR was achieved by 5/17 (29%) patients.

**FIGURE 2:**
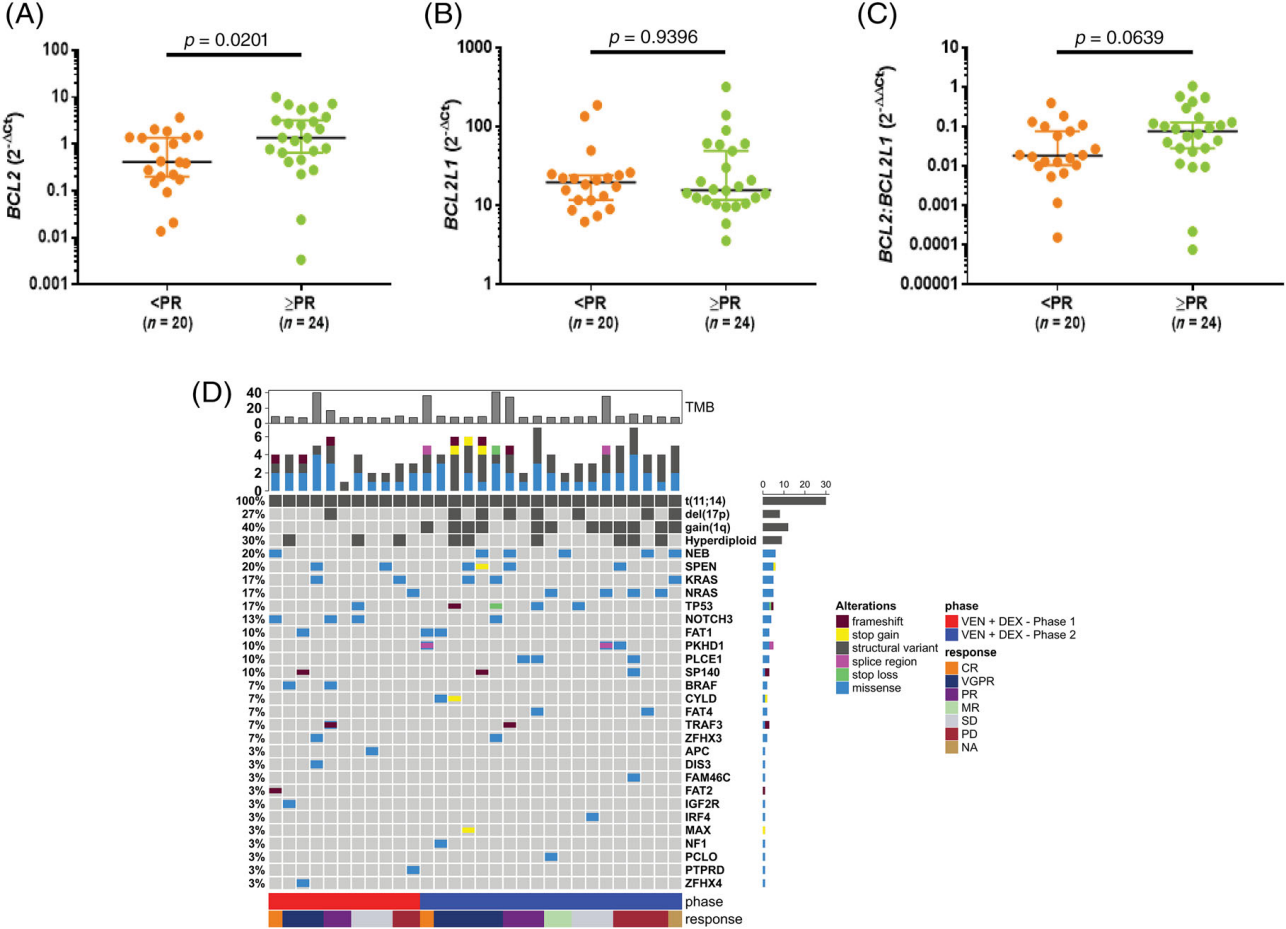
**Baseline *BCL2*, *BCL2L1*, and** ***BCL2*** **: *BCL2L1*** **gene expression levels by response in t(11;14) positive R/R MM patients treated with VenDex**. Quantitation of *BCL2 and BCL2L1* was performed on CD138‐selected BMMCs collected at baseline using qPCR. Presented are the (A) *BCL2*, (B) *BCL2L1*, and (C) *BCL2: BCL2L1* gene expression levels by response in t(11;14) positive R/R MM patients treated with VenDex. Horizontal bars represent the median and whiskers extend to the 95% confidence intervals. *p* values determined by Wilcoxon rank‐sum test. (D) Mutational landscape of t(11;14) positive patients treated with VenDex. Oncoprint representation of the frequency and characteristics of recurrent mutations in MM as determined by whole‐exome sequencing of CD138‐selected BMMCs collected at baseline from t(11;14) positive patients treated with VenDex. Colored squares indicate mutated genes, while gray squares indicate non‐mutated genes. Each color represents a different type of mutation: missense (blue), frameshift (red), stop codon gain (yellow), stop codon lost (green), splice region (pink). The structural variants t(11;14), del(17p), gain(1q), and hyperdiploid (gains in Ch5, Ch9 or Ch15) as determined by FISH are also denoted (dark gray). Percentages in the heatmap represent the mutation rate among all patients presenting at least one mutated gene of the reported gene list. Tumor mutation burden calculated by the number of non‐synonymous somatic mutations (single nucleotide variants and small insertions/deletions) per megabase in coding regions is shown on top of oncoprint. Study phase (Phase one: red; Phase two: blue) and best objective response (CR: orange; VGPR: blue; PR: purple; MR: teal; SD: gray; PD: red; NA: gold) are shown below the oncoprint

Baseline bone marrow aspirate samples were also evaluable for *BCL2* and *BCL2L1* gene expression by qPCR in 17/20 (85%) and 27/31 (87%) patients in phase one and phase two cohorts, respectively. A broad range of *BCL2* (median 2^‐ΔCt^: 0.93 [range: 0.003 to 9.918], *BCL2L1* (median 2^‐ΔCt^: 17.388 [range: 3.554 to 319.573], and *BCL2:BCL2L1* (median 2^‐ΔΔCt^: 0.0429 [range: 0.0001 to 1.0644] expression were observed, which were comparable between phase one and phase two cohorts. Consistent with the Ven mechanism of action in t(11;14) R/R MM, higher *BCL2* levels were observed in patients who achieved a PR or better (median 2^‐ΔCt^: 1.361 vs 0.4162; *p* = .0201) (Figure 2A). No association was observed between *BCL2L1* or *BCL2:BCL2L1* gene expression and response (Figure 2B,C). In addition, no difference in the median TTP was observed for patients with *BCL2*, *BCL2L1*, and *BCL2:BCL2L1* expression above or below the median qPCR value (Figure S3).

Whole‐exome sequencing for somatic mutations was performed in 11/20 (55%) and 19/31 (61%) patients in phase one and phase two cohorts, respectively. The mutational landscape of the biomarker evaluable patients was highly heterogeneous (Figure 2D). The mutation of genes involved in the MAPK pathway was most prevalent (13/30 pts, 43%). In the MAPK pathway, the most frequently mutated genes were *KRAS* (17%), *NRAS* (17%), *BRAF* (7%), and *NF‐1* (3%). Mutations in this pathway were mutually exclusive, and response rates to VenDex treatment were comparable to the overall study population (46% ORR; 39% ≥ VGPR). Recurrent mutations in the NF‐kB pathway were detected with inactivating mutations in *TRAF3* or *CYLD* observed in 4/30 (13%) patients. All four patients with a NF‐kB pathway mutation achieved a response (two VGPR, two PR).

Mutations in the *TP53* tumor suppressor gene were observed in 5/30 (17%) patients. The *TP53* mutations were detected in 3/7 (43%) patients with concurrent 17p deletion as detected by FISH, indicating bi‐allelic *TP53* inactivation. Response to VenDex treatment was observed in 2/3 patients (one VGPR, one PR) with concurrent 17 deletion and *TP53* mutation. While the somatic mutations identified were highly heterogeneous and subclonal, there were more patients with a high tumor mutation burden (TMB), defined as ≥20 mutations per megabase of sequenced DNA, in the phase two cohort (*n* = 4) compared to phase one (*n* = 1). Although a limited number of patients with high TMB were evaluable, response to VenDex was observed in four of the five patients with high TMB (one CR, two VGPR, one PR). No mutations in the *BCL2* gene were detected.

## 4 DISCUSSION

For patients with MM who are heavily pre‐treated and are refractory to an IMiD, PI, and monoclonal antibodies (eg, anti‐CD38), there are very few options available for treatment.^23^ The differentiated biology of t(11;14) positive MM with respect to BCL‐2 dependency offers an opportunity for a targeted therapeutic approach based on BCL‐2 inhibition. As previously reported, single‐agent Ven demonstrated promising anti‐myeloma activity in heavily pre‐treated patients with t(11;14) positive R/R MM, who had previously received a median of five prior lines of therapy.^13^ Dexamethasone, which is known to enhance the activity of anti‐myeloma therapies, can also increase the dependency of MM cells on BCL‐2 for cell survival and their sensitivity to Ven. The current study evaluated if the addition of Dex could improve clinical outcomes with Ven in t(11;14) positive R/R MM, including patients who had also failed therapy with an anti‐CD38 monoclonal antibody. This study showed promise for the VenDex combination treatment for patients with t(11;14) positive R/R MM.

The treatment with VenDex was generally safe and well‐tolerated. All hematological toxicities were manageable. Commonly reported non‐hematological AEs were diarrhea and nausea that were managed by the standard of care. Only two deaths were reported due to treatment‐emergent adverse events in this unique population of t(11;14) positive patients who were treated with the VenDex combination. The phase three BELLINI study reported higher mortality rates in patients regardless of genetic mutation status, and the patients were treated with a combination of VenDex and the PI bortezomib. However, the mortality rates were lower among patients harboring t(11;14) translocations in BELLINI.^24^ This is suggestive that t(11;14) positive patients may derive the most benefit with VenDex treatment.^25^ Nonetheless, safety should be further evaluated among R/R MM patients with t(11;14) positive treated with VenDex.

Patients in phase two VenDex who had received a median of five lines of prior therapy achieved 48% ORR, and the responses were both rapid (median TTR, 0.7 months) and durable (median TTP, 10.8 months). Of note, patients from this study who were t(11;14) positive and treated with Ven monotherapy had a DoR of 9.7 months.^13^ With the addition of VenDex, we observed longer DoRs. Patients in the phase one VenDex cohort had a DoR of 12.4 months while the median DoR was not reached for patients in the phase two VenDex cohort, suggesting that durable responses may be achieved with VenDex treatment in this patient population.

The phase two HORIZON study that enrolled patients with a median of five (range 2–12) lines of prior therapy reported that 79% patients who were refractory to daratumumab achieved an ORR of 22% in an all‐comer population when treated with melflufen and Dex combination.^26^ Patients enrolled in the phase two VenDex cohort in our study included 87% patients refractory to prior daratumumab therapy. The ORR was 48% but the patients were all t(11;14) positive. Cross‐study comparisons are challenging due to differences in patient characteristics and sample size of the studies, however, we believe that the VenDex combination shows promising efficacy in this cohort of patients with t(11;14) positive R/R MM. This study also supports the utility of a biomarker‐based approach to therapy in R/R MM.

In contrast to prior observations with Ven monotherapy treatment,^13^ no significant association between response and baseline *BCL2L1* or *BCL2:BCL2L1* expression was observed in VenDex treated patients, however higher *BCL2* levels were found in patients who achieved a response which is consistent with the mechanism of action in t(11;14) positive MM. These findings are consistent with the biological rationale of dexamethasone‐induced “BCL‐2 priming” and the combination activity with Ven as demonstrated in preclinical studies.^14^, ^15^, ^16^, ^18^, ^19^

The MM patients with the high‐risk abnormality del(17p) and gain(1q) have reduced OS in MM.^27^, ^28^ In addition, bi‐allelic inactivation of the tumor suppressor gene *TP53*, has been linked to dismal outcomes in MM.^29^ We observed response to VenDex treatment in five of the 10 patients with del(17p), including two of the three patients with concurrent TP53 mutation and 17p deletion. These results are consistent with those observed in chronic lymphocytic leukemia, another B‐cell malignancy, where responses were independent of 17p deletion, TP53 mutation, and TP53 function.^30^

While gain(1q) is not currently considered high‐risk by the IMWG classification, several studies have demonstrated it as a poor prognostic marker even when patients are treated with newer active agents.^28^, ^31^, ^32^, ^33^ Furthermore, 1q21 is the chromosomal region that contains the MCL1 locus, a known resistance factor to activity in MM.^11^ Importantly, a response to VenDex treatment was observed in eight of the 17 patients with gain(1q), further supporting the biological rationale of the combination in t(11;14) MM.

High somatic mutation loads are associated with increased genomic instability, resistance to therapy, and decreased survival in MM.^34^, ^35^ The mutational landscape analyses demonstrated that more patients with high TMB were in the phase two cohort compared to phase one. This finding is consistent with greater tumor genomic instability in a more heavily pre‐treated R/R MM population.^36^, ^37^ Similarly, more patients with del(17p) and gain(1q) were in the phase two cohort compared to phase one. Response to VenDex was independent of concurrent del(17p) or gain(1q), and high TMB status. However, our sample size was limited, and additional analyses are warranted. Importantly, response to VenDex was independent of mutations in key oncogenic signaling pathways in MM, including MAPK and NF‐kB.

In conclusion, VenDex demonstrated efficacy and manageable safety in patients with t(11;14) positive R/R MM. These results support further investigation of Ven combinations in this patient population, and the VenDex combination is being further investigated in an ongoing phase three trial (NCT03539744) in patients with t(11;14) positive R/R MM.

## CONFLICT OF INTEREST

JL Kaufman: Consultant for BMS, Janssen and Data Safety Monitoring Board for TG Therapeutics.

C Gasparetto: Honoraria from Janssen, BMS, Celgene; Consultant for Janssen, BMS, Celgene; Research support from Celgene; Travel, accommodations, or other expenses paid or reimbursed by Janssen, BMS, Celgene.

FH Schjesvold: Adboards for Amgen, Celgene, Takeda, Janssen, Oncopeptides, MSD. Honoraria from Amgen, Celgene, Takeda, Janssen, Novartis, SkyliteDX.

P Moreau: Honoraria and advisory boards for AbbVie, Janssen, Celgene/BMS, Amgen.

C Touzeau: Advisory board member for AbbVie, Celgene, Janssen, Takeda, Novartis, Amgen.

Research funding from AbbVie.

T Facon: No relevant conflicts to disclose, investigator in AbbVie funded clinical trial.

LH Boise: Advisory board member for Genentech, Research funding and honoraria from AstraZeneca.

S Unger, T Macartney, JA Ross, J Pesko, AH Salem, X Yang, F Dunbar, D Vishwamitra, Y Yu: Employees of AbbVie and may own stock.

WJ Hong: Employee of Genentech and owns Roche stock and options.

Y Jiang: Employee of Genentech and holds Roche stocks.

S Kumar: Research support to an institution for clinical trials from Celgene, Millennium/Takeda, Onyx, AbbVie, Janssen, Sanofi, Novartis; Consultant (with no personal compensation) to Celgene, Millennium, BMS, Onyx, Janssen, Noxxon; Honorarium from Skyline.

PC Maciag: is a former employee of AbbVie, currently employed by BMS, and may hold AbbVie stock.

JM Pauff: is a former employee of AbbVie and currently employed by Sarah Cannon Research Institute.

Venetoclax is being developed in collaboration between AbbVie and Genentech. AbbVie and Genentech funded this study (NCT01794520) and participated in the study design, research, analysis, data collection, interpretation of data, reviewing, and approval of the publication. All authors had access to relevant data and participated in the drafting, review, and approval of this manuscript. No honoraria or payments were made for authorship.

## AUTHOR CONTRIBUTIONS

Study conception and design: P.M., J.P., A.H.S.

Provision, collection and assembly of data: All authors contributed to data collection.

Data analysis and interpretation: All authors had access to the data and participated in data collection and interpretation.

Manuscript writing, editing, and approval: All authors.

## Supporting information


**Figure S1.** Study design and patient enrollment

**Figure S2.** Infection rates in Phase 1 and Phase 2 VenDex cohorts

**Figure S3.** Time to progression by a) *BCL2*, *b) BCL2L1*, and *c) BCL2:BCL2L1* expression

## Data Availability

AbbVie is committed to responsible data sharing regarding the clinical trials we sponsor. This includes access to anonymized, individual, and trial‐level data (analysis data sets), as well as other information (eg, protocols and Clinical Study Reports), as long as the trials are not part of an ongoing or planned regulatory submission. This includes requests for clinical trial data for unlicensed products and indications. This clinical trial data can be requested by any qualified researchers who engage in rigorous, independent scientific research, and will be provided following the review and approval of a research proposal and Statistical Analysis Plan (SAP) and execution of a Data Sharing Agreement (DSA). Data requests can be submitted at any time and the data will be accessible for 12 months, with possible extensions considered. For more information on the process, or to submit a request, visit the following link: https://www.abbvie.com/our-science/clinical-trials/clinical-trials-data-and-information-sharing/data-and-information-sharing-with-qualified-researchers.html.
