# Supplementary figures and images for: Targeting BCL‐2 with venetoclax and dexamethasone in patients with relapsed/refractory t(11;14) multiple myeloma
Source: Am J Hematol. 2021 Jan 19;96(4):418–27. doi: 10.1002/ajh.26083 (PMC7986778; doi:10.1002/ajh.26083)

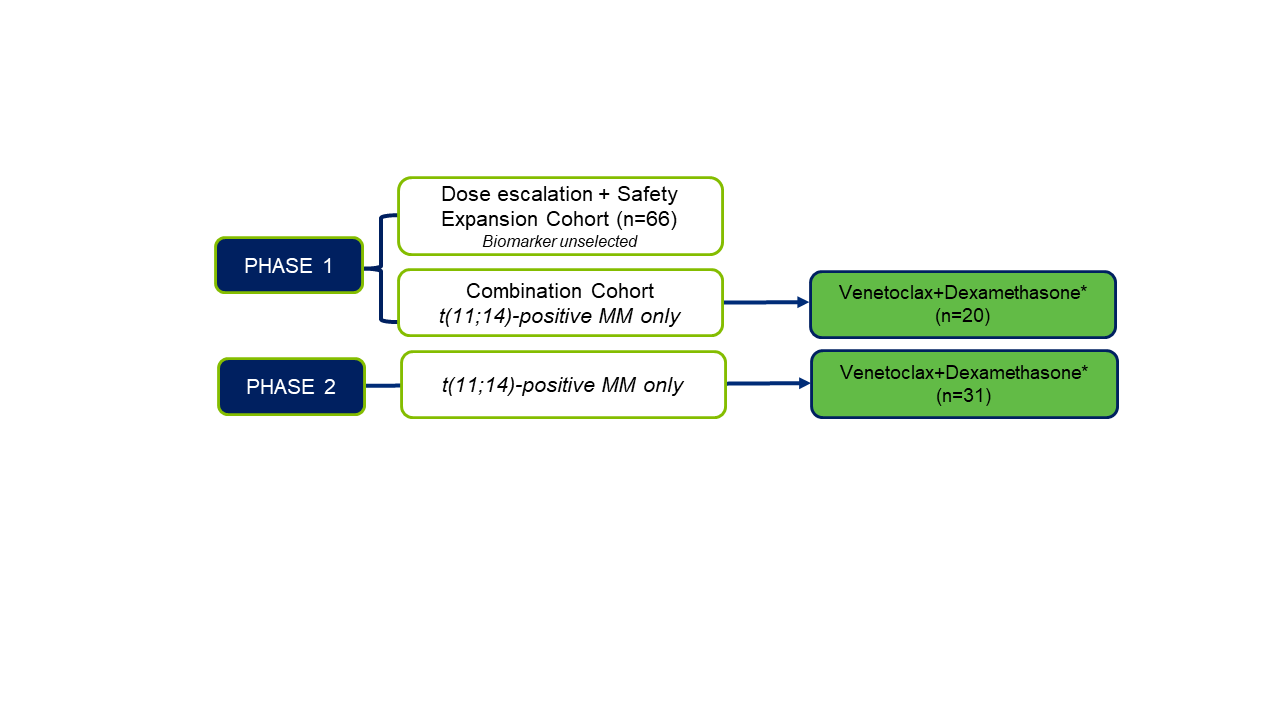

Supplement: Supplementary file 1 — Figure S1. Study design and patient enrollment [file AJH-96-418-s002.tif]

Supplemental Figure 2

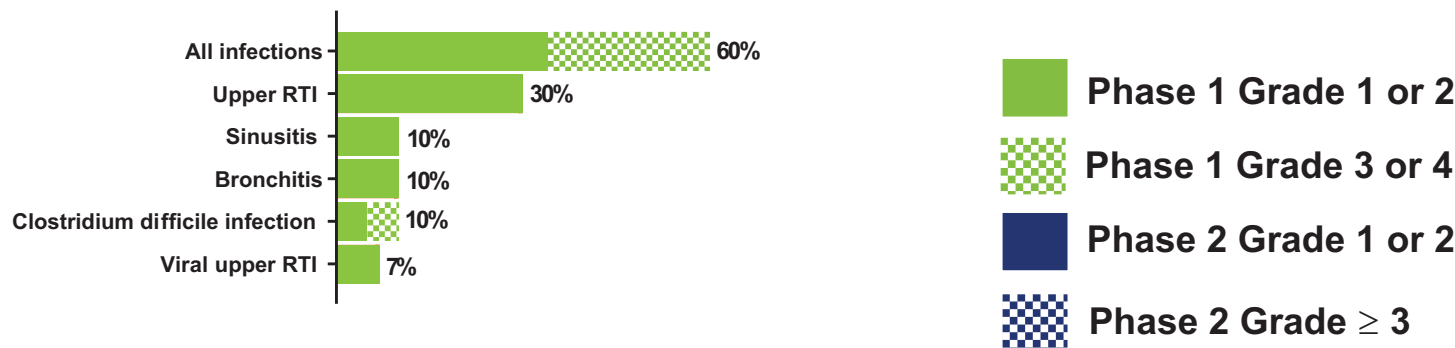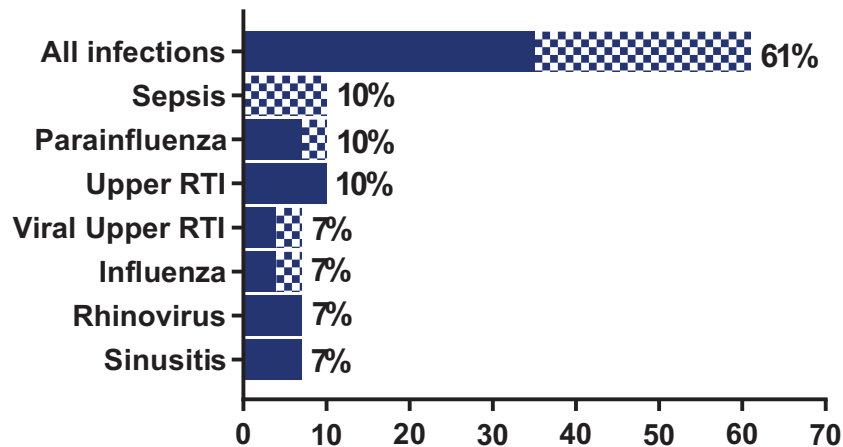

Supplement: Supplementary file 2 — Figure S2. Infection rates in Phase 1 and Phase 2 VenDex cohorts [file AJH-96-418-s001.pdf]

a

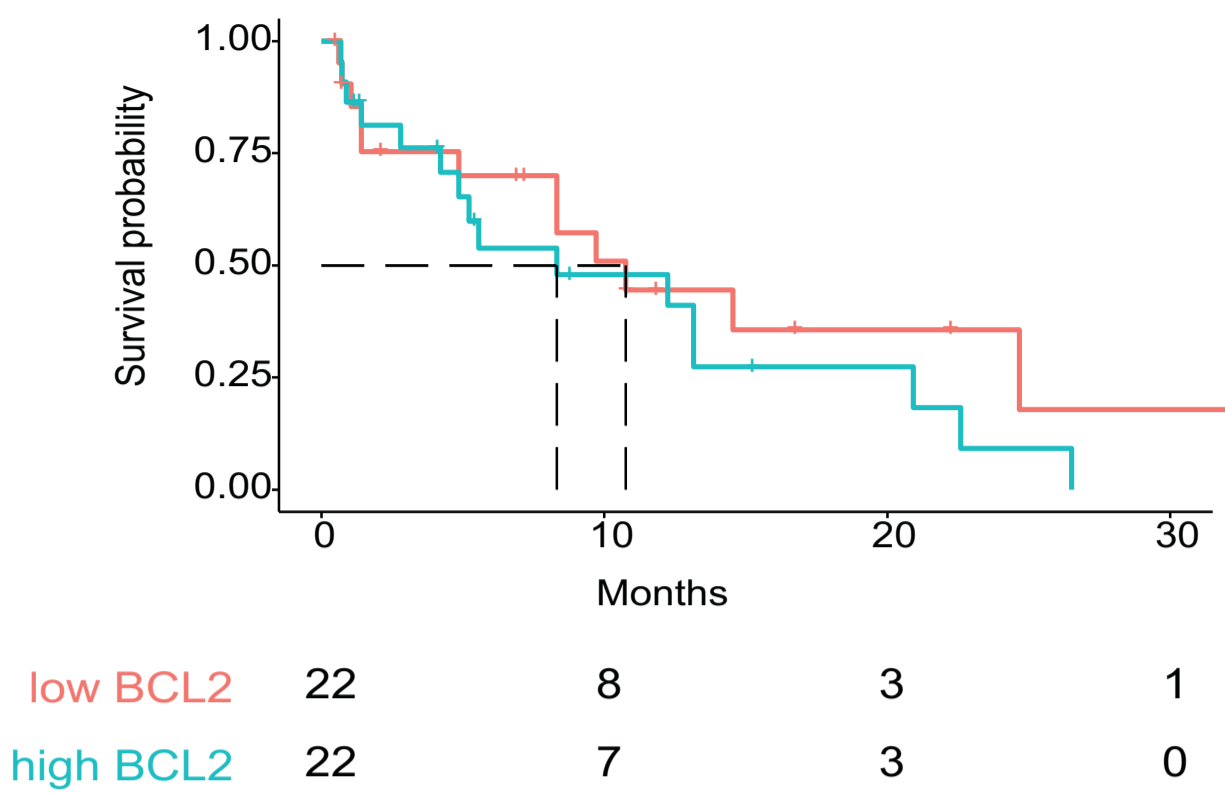

b

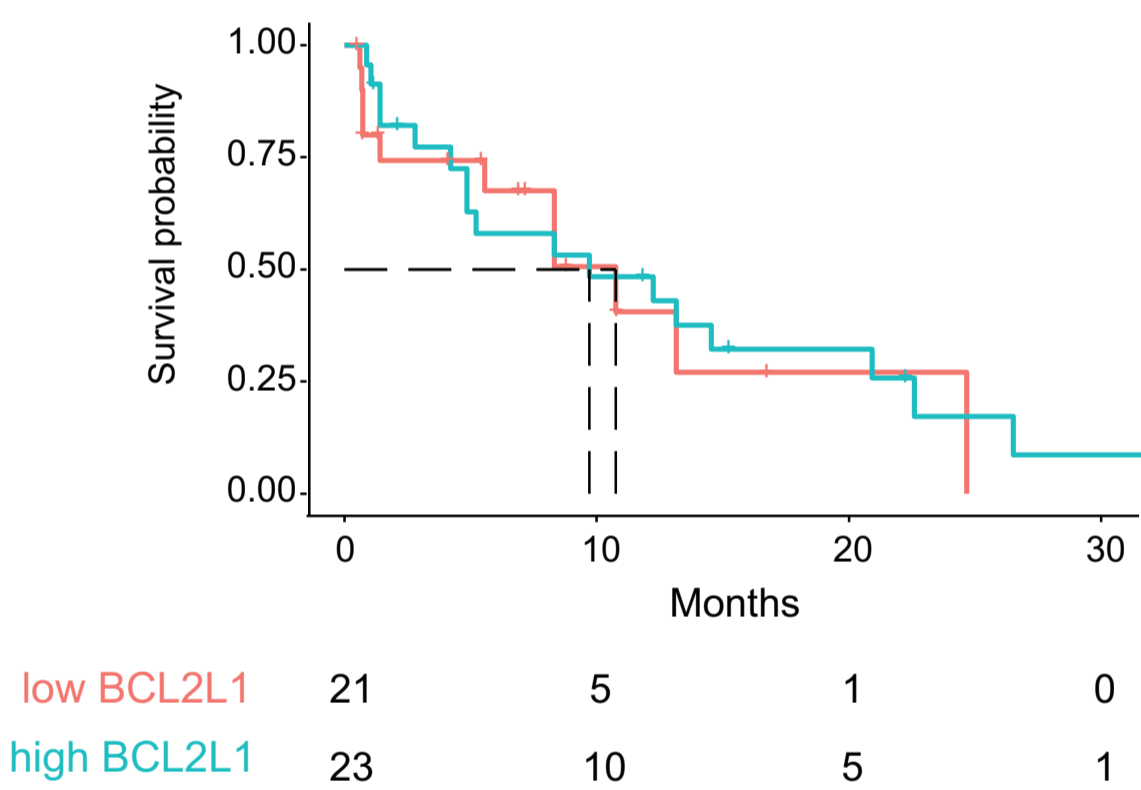

c

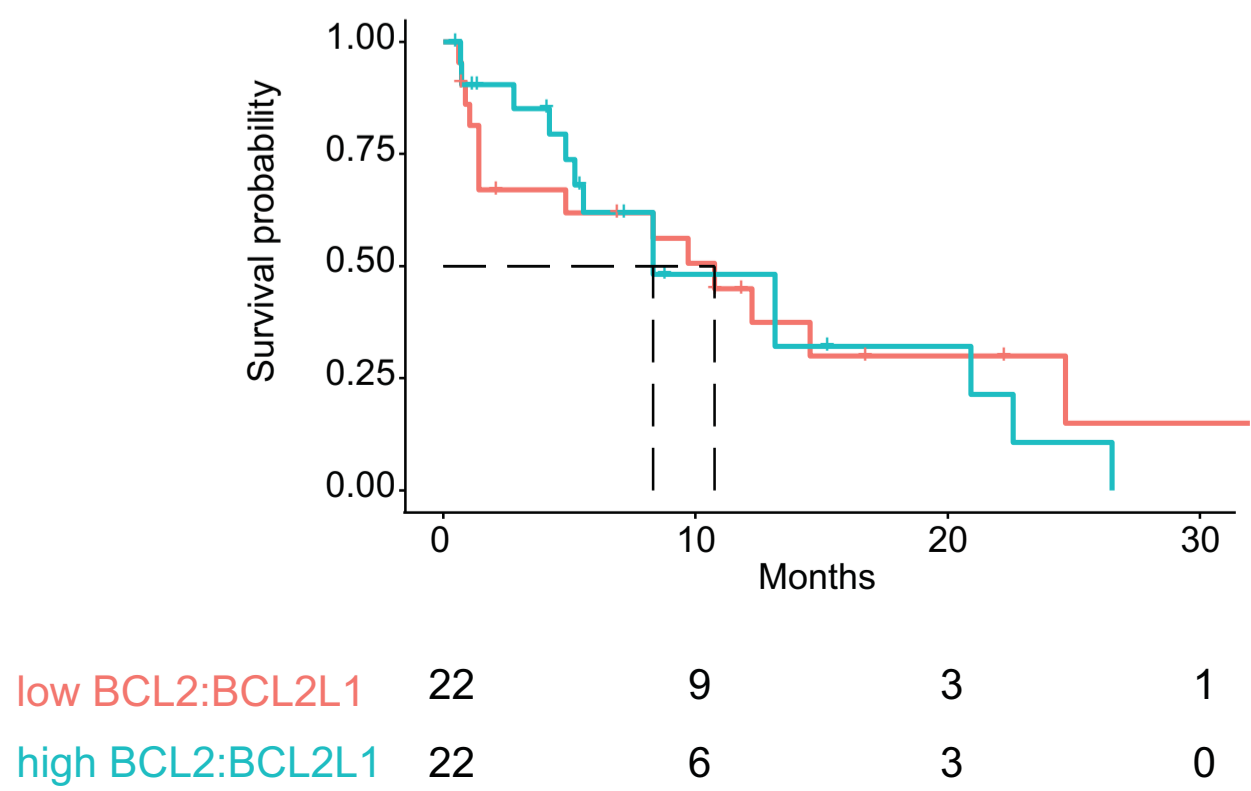

Supplement: Supplementary file 3 — Figure S3. Time to progression by a) BCL2, b) BCL2L1, and c) BCL2:BCL2L1 expression [file AJH-96-418-s003.pdf]
